# Delayed Infection of a Lymphocele following RARP in a Patient with Nonspecific Symptoms

**DOI:** 10.1155/2017/3935082

**Published:** 2017-03-15

**Authors:** Tomoki Taniguchi, Yoshito Takahashi, Mitsuhiro Taniguchi, Toru Yamada, Kenichiro Ishida

**Affiliations:** Department of Urology, Gifu Prefectural General Medical Center, 4-6-1 Noishiki, Gifu 500-8717, Japan

## Abstract

Pelvic lymphoceles are an infrequent complication after pelvic surgery and develop shortly after the surgery in most cases. We experienced a case of delayed infection of a lymphocele 6 months after robot-assisted radical prostatectomy (RARP) and pelvic lymphadenectomy. In this case, antimicrobial chemotherapy and percutaneous drainage were effective, and there was no recurrence of the disease. Most urologists do not recognize that infected lymphoceles can develop a long time after surgery; thus, infected lymphoceles should be kept in mind in patients with nonspecific infectious symptoms, regardless of the length of time after surgery.

## 1. Introduction

Lymphocele development is known as an infrequent complication after radical prostatectomy and pelvic lymphadenectomy [1]. Most lymphoceles develop shortly after the surgery and do not become symptomatic, though some can cause such problems as pain, deep vein thrombosis, pulmonary embolism, and infection. However, asymptomatic lymphoceles can be infected several months after the surgery. Most cases of delayed infection of lymphoceles complained of no specific symptoms. Therefore, its diagnosis is usually delayed. In addition, most urologists do not recognize that infected lymphoceles can develop several months or more than 1 year after radical prostatectomy and lymphadenectomy. There have been only 4 published reports of 7 patients with delayed infection of lymphoceles after the surgery: 3 cases in which the pathogenic bacteria are* Staphylococcus aureus*, 1 case with* S. agalactiae*, and 3 cases in which the pathogenic bacteria are not mentioned [2].

## 2. Case Presentation

A 79-year-old Japanese man with a prostate-specific antigen level of 6.5 *μ*g/ml presented to our department. He was diagnosed as having localized adenocarcinoma of the prostate, Gleason 3+3, cT2c N0 M0. He underwent transperitoneal non-nerve-sparing robot-assisted laparoscopic radical prostatectomy (RARP) with pelvic lymph node dissection, and a pelvic drain was placed for 3 days. The pathologic examination revealed that the tumor was pT2c with Gleason 3+5, and none of the dissected lymph nodes were malignant. On the 7th day after surgery, he was discharged from hospital with no complications. Six months after the operation, he visited a primary care doctor complaining of fever and fatigue. For a week, his symptoms continued. He was referred to the general medicine department in our hospital. A pelvic computed tomography (CT) scan showed a pelvic cyst, and a blood test revealed an abnormally high inflammatory reaction; he was then sent to our department and admitted.

**Figure 1:**
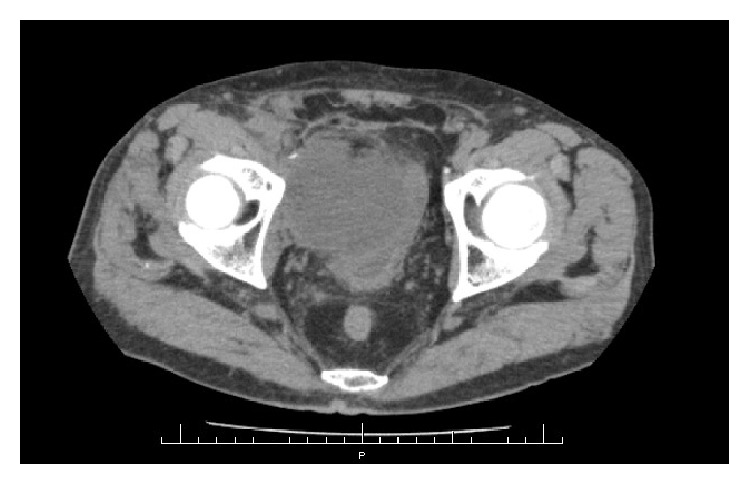
CT scan shows an 80 mm cystic lesion displacing the urinary bladder 6 months after the surgery.

On admission, his body temperature was 39.3°C. On physical examination, any specific findings, excluding right lower abdominal pain, were not observed. Laboratory data revealed a CRP of 22.38 mg/dl and WBC of 12600/*μ*l. A urine test showed no abnormal findings. Abdominal ultrasonography (US) and pelvic CT scan revealed an 80 mm cystic lesion displacing the urinary bladder (Figure 1).

**Figure 2:**
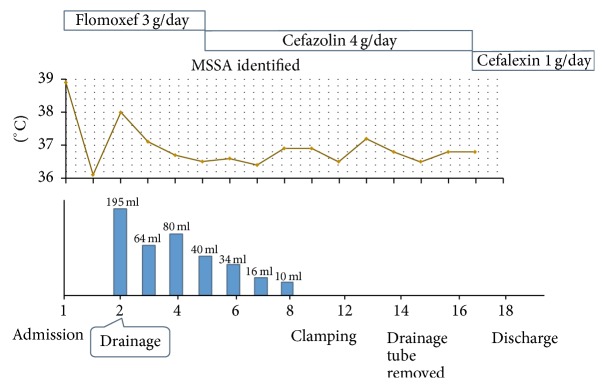
The treatment course.

**Figure 3:**
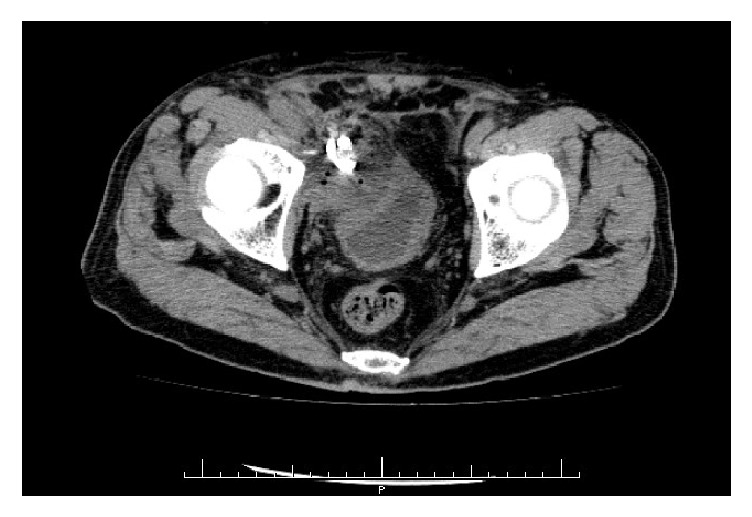
CT scan shows reduction of the lymphocele few days after clamping.

The treatment course is shown in Figure 2. We began treatment with intravenous administration of 3 g flomoxef per day. On the second day after admission, we performed US-guided drainage of the fluid collection and aspirated purulent fluid. Gram staining of the fluid showed Gram-positive cocci. Three days after drainage, we changed medication to cefazolin 4 g per day because methicillin-susceptible* S. aureus* was detected in the culture of the fluid. Seven days after drainage, his drainage gradually decreased to 10 ml, so we clamped the drainage tube. A few days after clamping, a CT scan showed reduction of the lymphocele (Figure 3), and then we removed the drainage tube. He was discharged from our hospital with treatment of cephalexin 1 g per day until the 15th day after drainage. A CT scan taken 2 months after discharge showed that the lymphocele had resolved. He has experienced no recurrence since then.

## 3. Discussion

Naselli et al. showed a 30% incidence of asymptomatic lymphocele after prostatectomy regardless of surgical procedures, open surgery, or laparoscopic surgery [3]. Some study also reported that symptomatic lymphoceles developed in 2–5% of cases undergoing either open surgery or RARP [1].

The present case did not have risk factors already known for lymphocele, such as diabetes, number of lymph nodes removed, extraperitoneal approach, and the use of low molecular weight heparin, which were previously reported as factors predictive of lymphocele formation by Raheem et al. [4]. In addition, our patient did not have particular characteristics similar to those of the other cases of prostatectomy.

Keskin et al. [5] reported a quite high incidence of lymphoceles within 1 month after RARP when performing US follow-up after RARP; most of the lymphoceles (76%) had disappeared by 3 months. However, a significant number (64%) of the lymphoceles that did persist over 3 months after RARP became symptomatic. Therefore, they recommended that routine US imaging be performed during the first 3 months after surgery. When a lymphocele is detected by the US examination, percutaneous drainage should be considered.

## 4. Conclusion

As many cases of prostatectomy are performed with RARP, the number of the delayed infections of lymphoceles would be increasing. Therefore, we should be aware of such a rare complication when a patient that underwent RARP with lymph node dissection presents with fever or other nonspecific symptoms.
